# Supercritical carbon dioxide: a solvent like no other

**DOI:** 10.3762/bjoc.10.196

**Published:** 2014-08-14

**Authors:** Jocelyn Peach, Julian Eastoe

**Affiliations:** 1 School of Chemistry, University of Bristol, Cantock’s Close, Bristol, BS8 1TS, U.K.

**Keywords:** CO_2_ chemistry, microemulsion, self-assembly, supercritical CO_2_, surfactant, viscosity

## Abstract

Supercritical carbon dioxide (scCO_2_) could be one aspect of a significant and necessary movement towards green chemistry, being a potential replacement for volatile organic compounds (VOCs). Unfortunately, carbon dioxide has a notoriously poor solubilising power and is famously difficult to handle. This review examines attempts and breakthroughs in enhancing the physicochemical properties of carbon dioxide, focusing primarily on factors that impact solubility of polar and ionic species and attempts to enhance scCO_2_ viscosity.

## Introduction

In this day and age, sustainability and renewability are watchwords. This includes focus within the scientific community on the philosophy of green chemistry, a concept encouraging the design of chemically efficient products and processes that reduce or eliminate the use or generation of hazardous substances [1]. This can, in theory, be achieved through the application of a set of ‘principles’, including reduced use and production of toxic reagents and products, avoidance of auxiliary substances where possible, and minimization of the energy requirements needed for the process, under this umbrella [2]. Attention has been drawn to the potential surrounding by the use of supercritical fluids and carbon dioxide in chemical processing and as a solvent [3], thus replacing the volatile organic compounds (VOCs) that are currently commonly used [4]. These VOCs are environmentally hazardous and notoriously difficult to dispose of so a reduction in use would improve the sustainability of many chemical processes. As well as being readily available, cheap, non-flammable, recyclable and unrestricted by the US Environmental Protection Agency (EPA) [3,5–15], supercritical CO_2_ (scCO_2_) is non-toxic so could potentially be used for the production of consumable products, such as pharmaceutical and food products [14] as well as already being an established system for numerous processes [16], including extractions [17], nanoparticle production and modification [18–21] and polymer processing [22–25]. Supercritical fluids also make appealing solvents due to the opportunities for tuning of solvent properties through changes in temperature and pressure; supercritical conditions are also easily reached with CO_2_, with critical pressure (*P*_c_) and temperature (*T*_c_) being 72.8 bar and 31.1 °C, respectively. Unfortunately, scCO_2_ suffers from a range of inconvenient physicochemical properties ordinarily required for an effective solvent, with a lower viscosity, dielectric constant [26–27] and surface tension in comparison to other common reference solvents. Also, as CO_2_ is a linear molecule with no net dipole moment there is significant difficulty dissolving polar and ionic species [26].

As well as looking to modify physicochemical properties of scCO_2_ to increase its appeal as a solvent, the ability to control properties will aid CO_2_ use in other avenues, including atmospheric CO_2_ capture, sequestration and storage as well as enhanced oil recovery processes (EOR) [28–29]. Increasing levels of atmospheric CO_2_ is a substantial challenge faced by scientists, politicians, engineers and economists, and carbon capture and sequestration (CCS) is one of the favoured techniques envisaged for tackling this. The ease and efficiency of both EOR and CCS require the fluid properties to be managed and controlled; both of these techniques would be both economically and technically more viable if the viscosity of CO_2_ used could be increased (leading to a reduction of viscous fingering during EOR and giving generally more overall control in CCS). The physicochemical properties of CO_2_ have thus far been managed through the addition of soluble and self-assembling additives, such as surfactants and polymers [11,30–51].

Viscosity of CO_2_ has been shown to be modifiable through the addition of aggregation and self-assembly of polymers and surfactants; however before internal/aggregated structures can be considered, and therefore viscosity modifiers developed, it is essential to ‘crack’ the puzzle of solubility, solvophilicity and CO_2_-philicity.

## Review

### Solvent compatibility – solubility and solvophilicity

Solubility is the property of a given substance (solute) that allows dissolution in a solvent leading to a homogeneous solution. It is measured in terms of the maximum amount of solute that can be dissolved in a solution at dynamic equilibrium, i.e., until the solution is fully saturated. Solubility can be quantified using either molar units, mol dm^−3^, or mass per unit volume units, such as g L^−1^. Solubility range can vary widely, from infinitely soluble (fully miscible) to poorly soluble. The term insoluble is often applied to solutes with very poor solubility, although in actuality there are very few truly insoluble systems. Solubility is determined by the balance of intermolecular forces between solvent and solute, along with the entropy change that occurs on dispersal of the solute, so is therefore dependent on both pressure, temperature and system polarity. Solvophilicity could be defined as the affinity a solute has for a given solvent, therefore CO_2_-philicity would represent the affinity a solute has for CO_2_.

A commonly coined term, ‘like dissolves like’ is often used in regard to solubility [52], i.e., polar solutes are easily solubilized by polar solvents. Molecular polarity arises from a separation of electrical charge within a molecule that leads to an electric dipole/multipole moment. It is dependent on a difference in electronegativity between the atoms within a compound and the degree of asymmetry in the compound. Point group determination is useful for the prediction of polarity. If individual dipole moments within a molecule cancel each other out, the molecule will not be polar. Any molecule with an inversion centre, a horizontal mirror plane (σ_h_), or multiple *C*_n_ axes will not have dipole moments and therefore will not be polar, (*C*_1_, *C*_s_, *C*_∞h_, *D*_∞h_ *C*_n_ and *C*_nv_ do not have a dipole moment). CO_2_ has a point group of *D*_∞h_, so is not polar.

As previously mentioned above, the difficulty observed in solubilisation of polar solutes in carbon dioxide is a substantial problem which must be overcome if the possibility of scCO_2_ as a solvent is to become a reality. Early studies by Consani and Smith [53] into the solubility of commercially available surfactants in scCO_2_ showed that the majority are insoluble, with a few non-ionic surfactants showing marginal solubility (out of the 130 or so surfactants tested). Following this discovery efforts have been directed towards the development of CO_2_-soluble additives. Focus has primarily been in surfactant and polymer design in water-in-CO_2_ (*w*/*c*) systems, however, attention is now beginning to expand into other alternative systems, including ionic liquid-in-CO_2_ systems [54–56] and the production of microstructures such as lamellar and bicontinuous phases [43,48].

### Solubility

#### Surfactants

**Surfactant tails – fluorinated surfactants:** Fluorine has a high electron affinity and electronegativity, hence fluorocarbons with a carbon number (*n*) that is equal to or greater than 4 have lower boiling points and refractive indices than the corresponding hydrocarbons [57] (different behaviour is observed when *n* < 4). Additionally, fluorocarbons have a larger molecular volume in the liquid state in comparison to corresponding hydrocarbons; therefore the polarizability per volume (α/υ) and the Hildebrand solubility parameter (δ, Equations 1) are significantly smaller than the equivalent hydrocarbons.

[1]
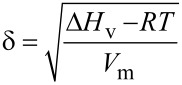


The Hildebrand solubility parameter δ can be used as an indication of solubility of a substance, as it provides a quantifiable estimate of the degree of interaction between materials. The parameters in Equation 1 are enthalpy change through vaporisation (Δ*H*_v_), gas constant R and temperature *T* divided by molar volume (*V*_m_).

As CO_2_ also has a low dielectric constant, α/υ and δ, it is expected that fluorocarbons and CO_2_ will be more compatible than HCs and CO_2_. It can therefore be said that fluorocarbons are more CO_2_-philic than the corresponding hydrocarbons. A range of surfactants are discussed throughout this review; the structures of which are displayed in Table 1.

**Table 1:** Structures of CO_2_-philic surfactants discussed in this review.

Compound	Structure	Shortened Term	Reference

**1**	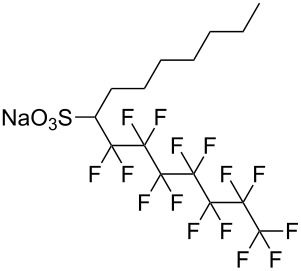	F7H7	[27,58]
**2**	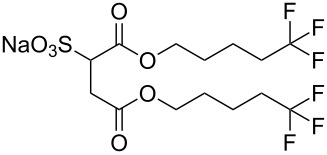	Di-CF1	[59]
**3**	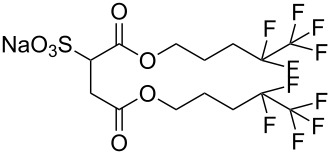	Di-CF2	[59]
**4**	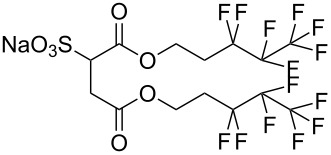	Di-CF3	[59]
**5**	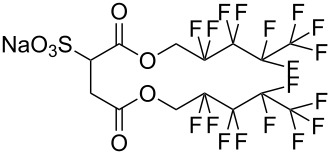	Di-CF4	[59]
**6**	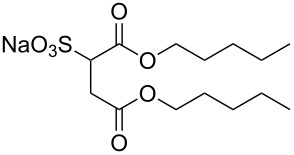	di-C5SS	[59]
**7**	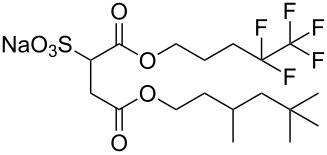	Hybrid CF2-AOT4	[59]
**8**	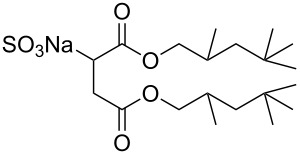	AOT3	[59]
**9**	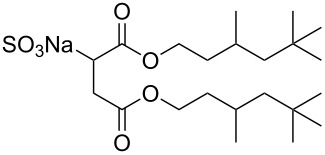	AOT4	[59]
**10**	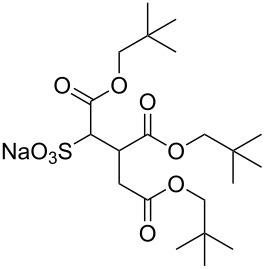	TC14	[60]
**11**	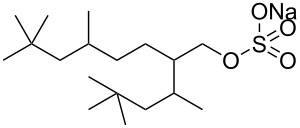	SIS1	[61]
**12**	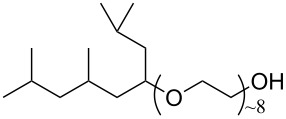	TMN-6	[61]
**13**	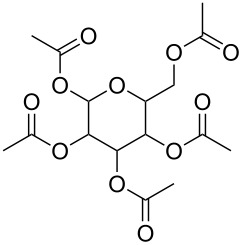	AGLU	[62]
**14**	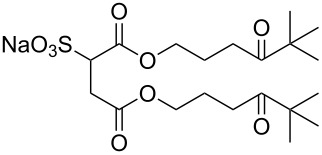	AOK	[37]
**15**	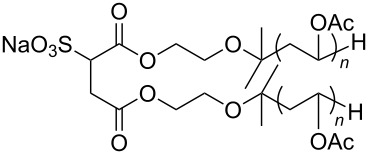	AO-Vac	[37]

Fluorocarbon-based surfactants have been used frequently in the field due to the high solubility of fluorocarbon chains in liquid and supercritical CO_2_. They were first introduced by Hoefling, Enick and Beckmann in 1991, through the synthesis of fluorinated AOT (Aerosol-OT, sodium dioctylsulfosuccinate) analogues and the observation of Windsor II microemulsions [26]. Microemulsions are thermodynamically stable dispersions of two or more immiscible/partially miscible fluids which are stabilised through the addition of amphiphilic molecules, such as surfactants or polymers. The domains created are on the nanometer scale leading to the dispersions being transparent or translucent in appearance. The microemulsion appearance does not alter over time. Following this discovery, a vast array of fluorocarbon surfactants have been investigated and successfully solubilised in dense CO_2_ to form *w*/*c* reverse micelles and microemulsions; this area has been extensively reviewed [36,38,63]. In more recent years, efforts have been made to reduce fluorocarbon use due to the high expense and ensuing environmental hazards [64–67]. Investigations focusing on partially fluorinated hybrid surfactants as a way to reduce fluorine content began in 1994 with the design and synthesis of F7H7, a partially fluorinated surfactant with an *n*-C7 fluorocarbon chain and an *n*-C7 hydrocarbon chain (Table 1, compound **1**) [27,58,68]. Subsequently both surfactant development and the understanding of applications of scCO_2_ have advanced considerably. Surfactant solubility has been previously expressed in a range of ways based on pressure and temperature phase behaviour studies. The phase instability *P*_trans_ (cloud point pressure) is the minimum pressure where a dispersion is a stable single transparent phase. It has been used as a measure of surfactant efficiency in CO_2_, with a lower *P*_trans_ being equated to a more efficient surfactant. The *w* value expresses the degree of water uptake or the number of water molecules solubilised by the additive/surfactant being tested in CO_2_ and is quantified by the water-to-surfactant ratio (Equation 2). A relationship between *w* value and *P*_trans_ has been highlighted, with *P*_trans_ increasing with increased *w* value [45].

[2]
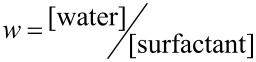


The effects of the surfactant chain length on microemulsion stability and the effects of fluorination have also been investigated, with studies showing that longer surfactant tails lead to increased stability in microemulsions. This is possibly due to increased interfacial activity and the changing of the terminal group from -CF_3_ to -CF_2_H reduces ease of microemulsion formation [35,69–70]. A systematic study by Mohamed et al. [59] identified the minimum amount of fluorine needed in a surfactant to remain CO_2_-philic. This was achieved with a controlled range of fluorinated AOT analogues, di-CF*n*, where *n* = 1–4 (with di-CF4 having fully fluorinated C4 groups and di-CF1 having the minimum level of fluorination, Table 1, compounds **2**–**5**), and a HC control analogue (di-C5SS, Table 1, compound **6**) containing no fluorine. The study also looked at the effects of replacing a terminal fluorine atom with hydrogen (*n*-CF_3_ to *n*-CF_2_H), hereby introducing a dipole moment at the end of the chain; a property that has strong possibility of decreasing CO_2_-philicity. This also leads to a reduction in the levels of fluorine, making the precursors cheaper [61,71]. Overall the study identified important factors for CO_2_-philicity as well as several avenues that could potentially be pursued further. A key CO_2_-philicity indicator identified was surfactant coverage at the water-CO_2_ interface, which can be quantified using surface tension measurements of aqueous solutions and the interface coverage index Φ_surf_, Equation 3.

[3]
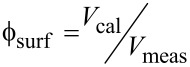


Where *V*_meas_ is the total fragment volume at the interface and *V*_cal_ is the volume of surfactants in total. *V*_meas_ is calculated using the area of surfactant headgroups (*A*_cmc_) and the interfacial thickness (τ) [59,72], Equation 4.

[4]
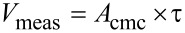


**Figure 1:**
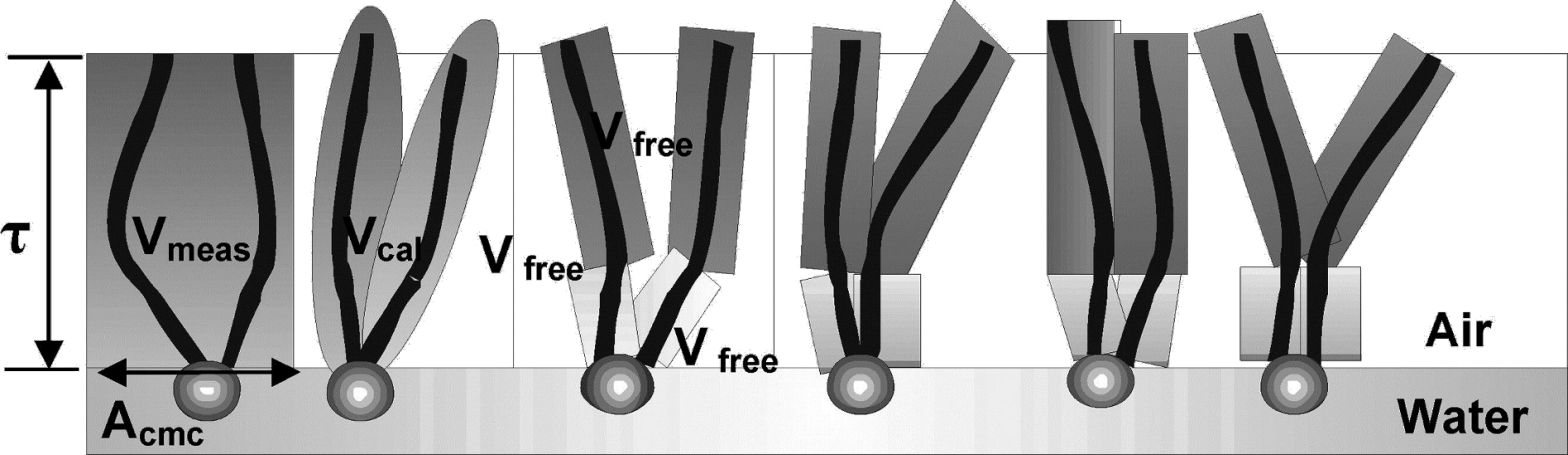
A visual representation of fluorocarbon surfactants at the air–water interface, highlighting the fragment and interfacial volumes is used to calculate the surface coverage (Φ_surf_). The measured surfactant molecular volume is *V*_meas_ and the calculated volume is *V*_cal_, based on the summation of fragments and the free space in the interface *V*_free_. Reprinted with permission from [72]. Copyright 2011 American Chemical Society.

An increased Φ_surf_ value ensured a greater separation at the interface between CO_2_ and water, see Figure 1.

**Figure 2:**
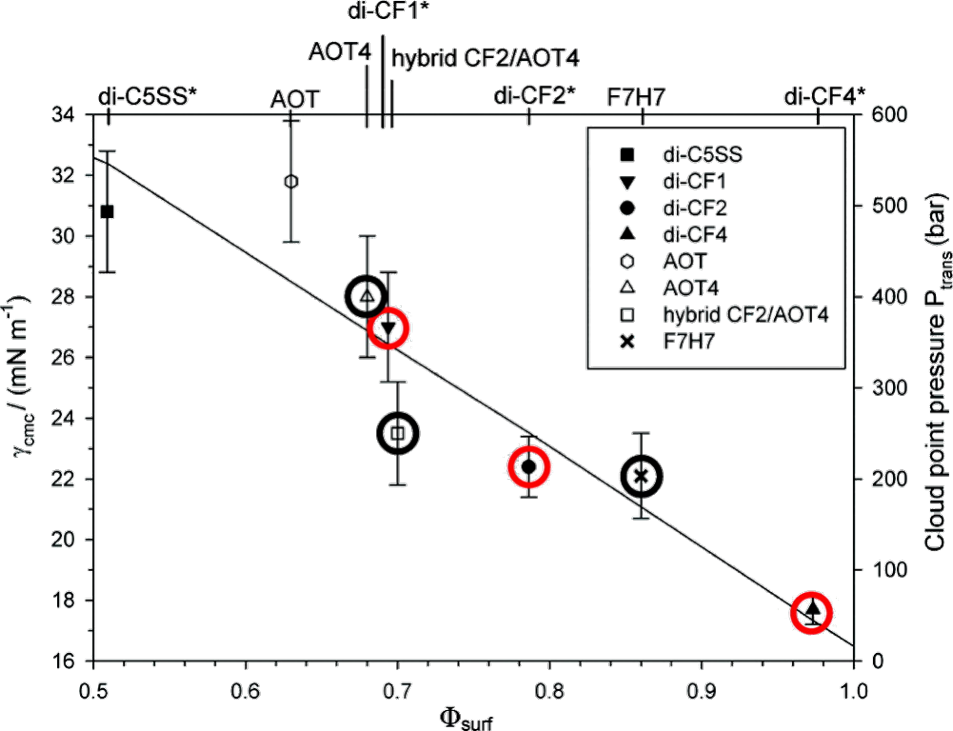
Correlation between relative surface coverage (Φ_surf_) – see Figure 1, limiting aqueous surface tension (γ_cmc_) and cloud point pressure (*P*_trans_). Circled points represent CO_2_-philic surfactants which are either fully fluorinated (red circles) or partially fluorinated (black circles), non-circled points represent non CO_2_-philic, hydrocarbon-based surfactants. There is a linear relationship between Φ_surf_, γ_cmc_ and *P*_trans_, with higher surface coverage values corresponding to lower cloud point pressure and surface tension at cmc. Reprinted with permission from [59]. Copyright 2012 American Chemical Society.

A linear relationship between Φ_surf_, *P*_trans_ and γ_cmc_ (surface tension of aqueous solutions) has been identified, with Φ_surf_ increasing with decreased *P*_trans_ and γ_cmc_. This has been observed with a range of surfactants, including fully and partially fluorinated and hydrocarbon-based surfactants (Figure 2).

**Surfactant tails – siloxane, hydrocarbon and oxygenated surfactants:** Though the use of fluorocarbons can be significantly reduced through the development of hybrid, semi-fluorinated surfactants, the race toward the development of a non-fluorinated, CO_2_ soluble surfactant is of primary focus in this field. These include hydrocarbon based, siloxane based and carbonyl based/oxygenated surfactants (Table 1, compounds **1**–**10**) [13,42,47,60–61].

Trisiloxanes have been identified as stabilisers for emulsions in both water-in-carbon dioxide and carbon dioxide-in-water systems but have yet to be seen to stabilise microemulsions. Investigations were carried out with a range of ethylene oxide (EO) repeat units, with an inversion of emulsion morphology from water-in-carbon dioxide to carbon dioxide-in-water as EO repeat units increased from 2 to 7. There was noteworthy stability at EO7 thought to be due to the strong solvation of the trisiloxanes by CO_2_ [47].

**Hydrocarbon based surfactants:** As previously mentioned, the compatibility of many commercially available hydrocarbon surfactants has been investigated to find that the majority are not CO_2_-philic [53], however, efforts to increase CO_2_-philicity have been made through intelligent surfactant design and synthesis. Several structural aspects have thus far been identified as increasing CO_2_-philicity of hydrocarbon surfactants, including the degree of surfactant tail branching and methylation [49], where increased tail branching and methylation led to increased solvophilicity in carbon dioxide in comparison to linear alkanes. This in turn led to the formation of *w*/*c* microemulsions with increased stability. It is thought that this is due to weaker interactions between surfactant tails as well as lower surfactant affinity to water thus leading to an improved partition coefficient.

Pitt et al. [73] drew attention to the fact that surfactants with *tert*-butyl chain tips have the lowest surface energies for hydrocarbon surfactants following a systematic study using a range of surfactant types. Following this study, two variations of AOT with *tert*-butyl tipped chains were observed to have low water-CO_2_ interfacial tensions (Table 1, compounds **8** and **9**). The presence of reversed micelles was also confirmed with small-angle neutron scattering (SANS) where the *w* value = 0, although phase separation occurred immediately upon water addition [59]. SANS is a non-invasive technique that utilizes elastic neutron scattering at small scattering angles in order to investigate internal sample structure over length scales of ~10 Å–1000 Å. It is used extensively in the field of colloid science due to the ability to identify a range of internal structures, as well as having the capacity to contrast individual components in a multicomponent mixture through selective isotropic (hydrogen/deuterium) labelling. Research around a group of tri-chain surfactants has been carried out through the addition of a third extensively methylated tail in hope to both enhance CO_2_ compatibility and to also reduce surface energy [39]. TC14 (sodium 1,4-bis(neopentyloxy)-3-(neopentyloxycarbonyl)-1,4-dioxobutane-2-sulfonate, Table 1, compound **10**) was solubilised in water, heptane and supercritical carbon dioxide and aggregates were characterised with SANS [39,60]. Results from surface tension measurements show that γ decreased as the number of tails increased, which is attributed to hydrocarbon tail packing efficiency balanced against headgroup repulsions, as well as the increase in the number of low energy methyl groups per headgroup. Expectations surrounding the impacts of chain-tip structure on CO_2_-philicity also supported data from previous studies [49], with structures having a greater extent of chain-tip branching showing a lower *P*_trans_ in comparison to those which baring less chain-tip branches [60]. Sagisaka et al. have synthesised and successfully solubilised a hydrocarbon based CO_2_-philic surfactant, SIS1 (sodium 2-(4,4-dimethylpentan-2-yl)-5,7,7-trimethyloctyl sulfate, Table 1, compound **11**) [61]. It was designed on the basis that a strongly hydrophobic tail was needed to efficiently solubilise surfactant in CO_2_ alongside the discovery that a highly methylated isostearyl unit is highly CO_2_-philic following the successful stabilisation of silver nanoparticles using isostearic acid [74]. It was compared to TMN-6 (Table 1, compound **12**), a non-ionic surfactant with highly branched alkyl tails and around eight oxyethylene units which has previously been reported as solubilising water up to a *w_o_* value of 30 (*w*_o_ value is similar to *w* value, however, uses a corrected water-to-surfactant ratio, where the number of moles of water solubilised by carbon dioxide is taken into account) when temperatures and pressures exceed 55 °C and 210 bar, respectively [75–77]. SIS1 has been observed to achieve a *w_o_* value of 50 but only with high temperature and pressure (55 °C and 210 bar). Both the cmc and the *A*_cmc_ (the surface area per molecule) of SIS1 were around 1.5 and 1.7 times larger than that of TMN-6 (1.5 × 10^−3^ mol L^−1^ cf. 8.8 × 10^−4^ mol L^−1^ and 103 Å^2^ cf. 70 Å^2^ respectively) resulting potentially from the bulkier isostearyl tail or from the increased electrostatic repulsion from the larger sulfate head group. The surface tension at cmc was marginally lower in SIS1 cf. TMN-6 (25 mN m^−1^ cf. 26 mN m^−1^, respectively), indicating that SIS1 has a greater ability to lower interfacial tension and therefore a higher solubilizing power in CO_2_. It is hypothesised that *w*/*c* microemulsions may be obtained at lower pressures and temperatures if; (i) the CO_2_-philicity of reversed micelles can be increased, (ii) if there are reduced attractive interactions between micelles or (iii) if the solvent power of CO_2_ could be tuned to make it more hydrocarbon-like [61]. It was reported that these properties could be modified through co-solvent/co-surfactant addition or by tuning the position and the amount of methyl groups on the surfactant tail [61].

**The concept of FFV:** A range of different models have been used to attempt to predict and quantify CO_2_-philicity. One very popular approach is the concept of fraction free volume, or FFV, defined by Johnston et al. [78]. The fractional free volume of an adsorbed monolayer is calculated from surfactant tail geometry and surface coverage, using the surfactant tail volume (*V*), the surfactant tail length (*t*) and the area of the surfactant headgroup at critical micelle concentration (*A*_cmc_), Equation 5:

[5]
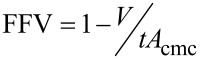


**Figure 3:**
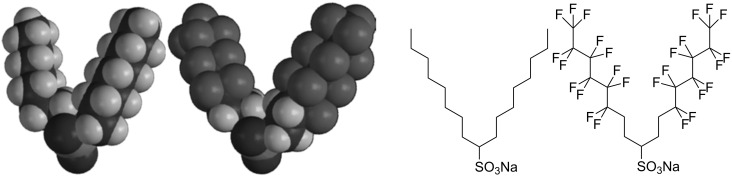
Structures illustrating the difference in fraction free volume (FFV) within a surfactant tail region through fluorination. On the left is a surfactant with a relatively high fractional free volume (DiH8) and on the right a surfactant with relatively low fractional free volume. The use of bulky fluorine atoms decreases the volume available to the solvent, hence reducing FFV value [78]. Reprinted with permission from [78]. Copyright 2004 American Chemical Society.

Surfactants with lower FFV values are expected to be effective CO_2_-philes, corresponding to improved solubility of surfactants and therefore increased stability with the formation of *w*/*c* microemulsions. The concepts and properties identified as effectively making surfactants soluble in CO_2_ are known to reduce FFV; fluorinated surfactants have a significantly larger tail volume in comparison to their hydrocarbon counterparts, and hydrocarbon surfactants with increased branching and a larger number of surfactant tails (tri-chain surfactants vs double chain surfactants) have been seen to be more CO_2_-compatible [39,49,60,73,78]. A reduced value for FFV leads to a more densely packed interfacial film and therefore a decrease in both CO_2_ and water penetration into the surface layers. Penetration of CO_2_ into the surfactant film could destabilise the aggregation structures, see Figure 3. Unfortunately the concept of FFV cannot be used exclusively as a measure of CO_2_-philicity but has served as a useful tool along with *P*_trans_ and Φ_surf_, for guiding selection and design of CO_2_-compatible surfactants.

**Oxygenated surfactants:** Oxygenated hydrocarbon chain surfactants have also been identified as potential replacements for fluorocarbon surfactants for applications in supercritical carbon dioxide. Kazarian et al. carried out a study investigating the interactions between CO_2_ and carbonyl groups using FTIR spectroscopy with polymers incorporating electron donating (carbonyl) groups. These polymers showed splitting of the band corresponding to the CO_2_ *v*_2_ mode, which was not observed in spectra from polymers without electron-donating groups [71]. The splitting is likely to be caused by Lewis acid–base interactions, arising from electron donation from the lone pair of the oxygen in a carbonyl group to the electron-deficient carbon atom in CO_2_. On this basis carbon dioxide should also interact favourably with other Lewis bases, another potential reason that fluorocarbon surfactants exhibit high solubility in scCO_2_ [26,79]. This is also supported by Reilly et al., who indicated that CO_2_ is more likely to behave as an electron acceptor over an electron-pair donor, through a study investigating CO_2_ interactions with *d*-methanol. Research confirmed that a *d*-methanol–CO_2_ complex occurred through Lewis acid–base interactions over hydrogen-bonding interactions [80]. Oxygenated hydrocarbon-based molecules have also been designed for use in CO_2_ by Raveendran et al., where selected carbohydrates were solubilised indicating the potential that they hold as alternative CO_2_-philic groups (Table 1, compound **13**) [62]. Eastoe et al. stabilised spherical reverse micellar microemulsions (characterised by SANS) with specifically designed AOT analogues; one containing a carbonyl group in each chain along with a *tert*-butyl at the chain tip (AOK, Table 1, compound **14**) and another with twin vinyl acetate oligomeric chains (AO-Vac, Table 1, compound **15**).

**Polymers:** Amphiphilic block-copolymers are classic examples of molecules which have an inherent ability to adsorb at interfaces and generate aggregation structures. Polymeric micelles may be generated in water and have been used in a range of applications, including biomedical science for drug delivery [81–82]. Judicial choice of the chemical nature of block components could help drive the formation of structures, such as reverse micelles, in scCO_2_. Many of the factors that have been employed to increase CO_2_-philicity of small molecule surfactants have reapplied during the design of CO_2_-philic polymers. The structures of polymers discussed in this review can be seen in Table 2.

**Table 2:** Structures of CO_2_-philic polymers discussed in this review.

Compound	Structure	Name	Reference

**16**	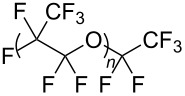	Krytox 16350	[83]
**17**	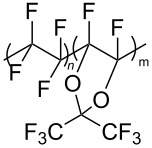	Teflon AF	[11–12]
**18**	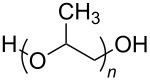	Poly(propylene glycol)-diol	[13]
**19**	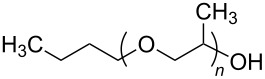	Poly(propylene glycol) monobutyl ether	[13]
**20**	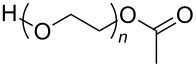	Poly(propylene glycol) acetate	[13]
**21**	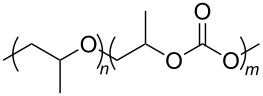	Poly(ether carbonate)	[13]
**22**	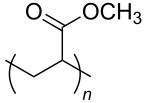	Poly(methyl acrylate)	[84]
**23**	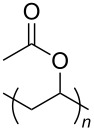	Poly(vinyl acetate)	[84]
**24**	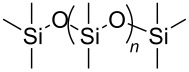	Poly(dimethylsiloxane)	[85–86]
**25**	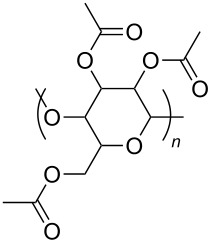	Cellulose triacetate	[87]
**26**	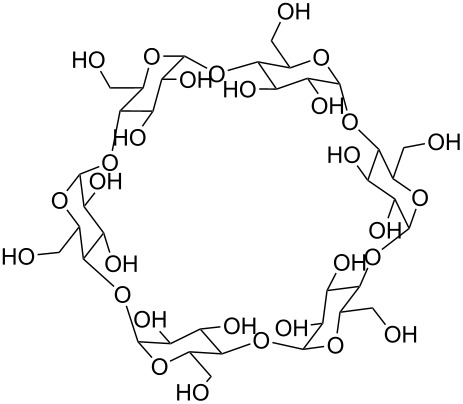	Cyclodextrin	[88]
**27**	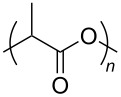	Poly(lactic acid)	[89]
**28**	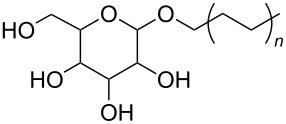	Glucopyranoside	[89]
**29**	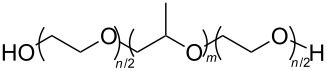	Pluronic	[51]

**Fluorinated polymers:** One of the first polymers reported to be solubilised in CO_2_ was Krytox 16350 (Table 2, compound **16**), a perfluoropolyether (PFPE) oil [83] with a molecular weight of 11350 g mol^−1^. The study showed that polymer solubility decreases with increased molecular weight with lower molecular weight polymers (<15000 g mol^−1^) showing high solubility in scCO_2_. The effects of head group composition and polymer chain length were investigated by Howdle et al. [90]. When the PFPE polymers were in their carboxylic acid form no microemulsions were formed, however, upon the conversion of the headgroups to ammonium carboxylates *w*/*c* microemulsions were observed. The length of the polymer tail was also shown to affect the ability to form microemulsions. The optimum molecular weight was found to be around 2500 g mol^−1^, with longer chain lengths being too CO_2_-philic, whereas shorter chain lengths were too hydrophilic to disperse extensive quantities of water within reverse micelles [90]. Partially fluorinated analogues, including an ethylene-based copolymer and fluorinated Teflon analogue Teflon AF have also been solubilised in CO_2_ (Table 2, compound **17**) [11–12].

**Non fluorinated polymers:** There has also been a large amount of research around non-fluorinated polymer analogues, for the same reasons as surfactants, to reduce fluorocarbon use. The identification of Lewis acid–base interactions as a mechanism for solubility in CO_2_ has given rise to many potentially CO_2_ soluble polymers, including oxygenated and acylated species [26,71,79–80]. The first non-fluorous polymers to be effectively designed and solubilised were poly(propylene glycol)-diol, poly(propylene glycol) monobutyl ether, poly(propylene glycol) acetate and poly(ether carbonate), (Table 2, compounds **18**–**21**) [13]. Beckman et al. developed a set of design rules for CO_2_-philic polymers based on both theoretical and experimental approaches [91]. These include (a) chain flexibility (for example, the addition of ether/oxygen linkages can increase the flexibility within the polymer backbone or side chains); (b) free volume, similar to the FFV principle mentioned earlier, with *tert*-butyl groups and other highly branched moieties giving a decrease in free volume; (c) presence of functional groups that have thermodynamically favourable interactions with CO_2_; (d) low crystallinity; this can be decreased by increased branching structures on side chains and finally (e) a low glass transition temperature (*T*_g_) [92]. There are also several factors that have been identified as having a negative impact on polymer solubility, including the presence of amine-functional groups and allyl polymers with a –CH_2_-spacer between the polymer backbone and the side chain or group [85,91]. These have since been used more as a set of guidelines as opposed to a set of hard-and-fast rules; poly(methyl acrylate) (PMA - Table 2, compound **22**) has a greater degree of acetylation than poly(vinyl acetate) (PVAc - Table 2, compound **23**) but has a much higher melting point and is insoluble in CO_2_ [84].

A significant amount of experimental and theoretical work has been carried out on the addition of CO_2_-philic moieties to polymer backbones and side groups, including the addition of tertiary amines and pyridine (all analogues tested were insoluble) [91] and siloxanes (poly(dimethylsiloxane) (PDMS, Table 2, compound **24**)), which has the highest solubility of all known non-fluorinated polymers in carbon dioxide [85–86]. There is also promise being seen with organic hydroxylated and oxygenated compounds such as cellulose triacetate [87], cyclodextrins [88], amorphous poly(lactic acid) and glucopyranoside [89] (Table 2, compounds **25**–**28**) as well as polymers with incorporated ether linkages [87].

**Figure 4:**
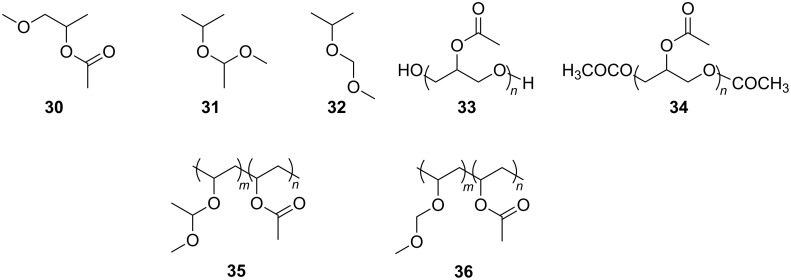
Structures of three CO_2_-philic candidates: (**30**) MIA, (**31**) 2MEP and (**32**) 2MMP and the respective polymer candidates they produce: (**33**) -OH terminated oligo(3-acetoxy acetate), OAO, based on MIA; (**34**) -OCOCH_3_ terminated OAO, also based on MIA; (**35**) PVMEE, poly(vinyl 1-methoxyethyl ether-co-acetate), based on 2MEP and finally (**36**) PVMME, poly(vinyl methoxymethyl ether-co-acetate), based on 2MMP.

Beckman and Enick et al. have a large body of both practical and theoretical work around polymer solubility in carbon dioxide [84]. The first reported use of ab initio modelling to design oxygenated hydrocarbon polymers for use in CO_2_ was in 2009, where one oligomer and two polymers were synthesised and found to be soluble in CO_2_ after ab initio predictions. The molecules OAO, PVMME and PVMEE were derived from the smaller CO_2_-philic moieties MIA, 2MME and 2MEE. It is hoped that other macromolecules could also be designed based on these moieties in time (Figure 4).

Unfortunately, ab initio modelling can be quite limiting as there are interactions and factors that cannot be taken into account. These include effects of temperature, density effects of the solvent, polymer–polymer interactions and the fact that only interactions between polymer segments and the solute can be computed, as the polymer cannot be represented as one macromolecule but instead must be represented in smaller fragments.

The induced micellisation by scCO_2_ of commercially available Pluronic polymers/poloxamers has also recently been observed at low temperatures [51]. Pluronics are non-ionic triblock copolymers with a central hydrophobic polyoxypropylene (PPO) unit edged by two hydrophilic polyoxyethylene (PEO) units (Table 2, compound **29**). It is hypothesised that the increased hydrophobic interaction of the internal PPO blocks with CO_2_ during addition is responsible for the change in morphology. These polymeric micelles differ slightly from those that have been previously reported due to the amphiphilic nature of their cores; ensuring that both non-polar and polar components could be dissolved in the micellar interiors.

#### Conclusions – factors impacting solubility in CO_2_

Significant effort has gone into further understanding the origins and intrinsic properties for both surfactant and polymer solubility in scCO_2_, which have been highlighted above. These include the inclusion of CO_2_-philic moieties which show favourable interactions with CO_2_, such as Lewis bases and fluorocarbons. Effort has been made to move away from the use of fluorocarbons in both surfactant and polymer design, but they are still potentially the most CO_2_-philic moieties observed thus far. This is thought to be due to their high molar volume and as well as their lower polarisability volume and solubility parameter, leading fluorocarbons to be more akin to CO_2_ (with low dielectric constant) than the respective hydrocarbon counterparts. There has been success in the design and synthesis of non-fluorinated polymers and surfactants, with hydrocarbon based, siloxane based and carbonyl and oxygenated hydrocarbon surfactants and polymers successfully solubilised [11–13,26,83–84,86,91].

Several models for quantifying solubility have also been developed: (A) the concept of Fractional Free Volume. Depending on surface tail geometry and surface coverage calculated using the tail volume and length and the surfactant headgroup, a lower FFV value is expected to be a better CO_2_-phile because of a more densely packed interfacial film, leading to increased stability at the interface. (B) Surfactant coverage at the interface (Φ_surf_). This is a similar property to FFV, but taking into account the fractional fragment volume of surfactant at the interface in respect to the volume of surfactant in the entire system. The fragment volume at the interface is calculated using the interfacial thickness and the area of surfactant headgroups at critical micelle concentration. Increased Φ_surf_ corresponds to greater separation between two phases at the interface.

### Viscosifiers for CO_2_

Though solubility of additives in CO_2_ is a very important factor, the eventual goal would be to have the ability to tune solvent properties with solutes, as is commonly done with regular solvents (e.g., water). As discussed above, a huge amount of research has been carried out around solubility and identifying and quantifying the properties that make any given additive CO_2_-philic. Alongside this, work focusing on modification of CO_2_ solvent properties has been undertaken; viscosity is one of these key properties, with viscosity enhancing solutes acting through increasing internal structure. The majority of structures observed with surfactants and polymers in CO_2_ have been spherical reversed micelles [69], however, other anisotropic surfactant aggregate structures have been observed. Surfactants and polymers that undergo self-assembly and aggregation provide convenient ways to develop structure and affect viscosity. Examples of self-assembly structures that are commonly used to enhance viscosity include ellipsoid and rod-like micelles, worm and lamellar structures, bicontinuous phases and also the formation of gels [32,46,49–50,93–95].

**Figure 5:**
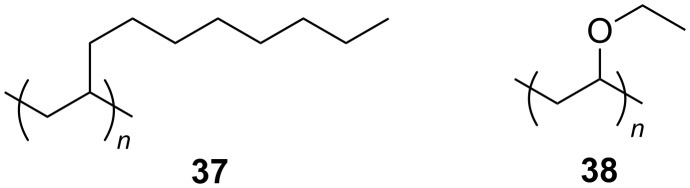
Structures of poly(1-decene) (37, P-1-D) and. poly(vinyl ethyl ether) (38, PVEE).

**Polymeric CO_2_ viscosifiers:** Several polymeric thickeners for CO_2_ have previously been identified, following investigations into polymer solubility in CO_2_ [96–97]. Heller et al. identified poly(1-decene) (P-1-D, Figure 5, compound **37**) as having potential CO_2_ viscosifying properties due to its high solubility in CO_2_ [97]. This work has been built on by Zhang et al. along with another low molecular weight, CO_2_-philic polymer; poly(vinyl ethyl ether), (PVEE, Figure 5, compound **38**) [98–99].

Viscosities of the polymer-thickened CO_2_ were measured by capillary viscometry across a range of pressures along with cloud point pressures. Higher *P*_trans_ values were observed for PVEE systems in comparison to the P-1-D systems, attributed to the presence of the oxygen containing ether group in the polymer (previously established as a group capable of increasing CO_2_-philicity and therefore solubility in CO_2_) [26,71,79–80]. Results showed that the viscosity of the polymer thickened CO_2_ (μCO_2_/PVEE) was around 13 to 14 times higher than that of the pure CO_2_ (μCO_2_) (μCO_2_ = 0.048–0.063 mPa s cf. μCO_2_/PVEE = 0.68–0.95 mPa s and μCO_2_/P-1-D = 0.70–0.93 mPa s) at 329.15 K [98]. Enick and Beckman et al. successfully enhanced the viscosity of dense CO_2_ by a factor of around 5 to 400 through the addition of fluoroacrylate and styrene copolymers at a range of polymer concentrations (1–5 w/w %) and styrene:fluoroacrylate molar ratios [100]. Cloud point pressures indicated that polymer solubility decreased with increased concentrations of styrene in the polymer chain, due to poor solvency of styrene in CO_2_. It was anticipated that π–π stacking between phenyl groups is a main contributor to the viscosity increase, through its provision of a fundamental intermolecular force needed to raise viscosity in a system. The optimum composition of polymers for viscosity enhancement in this study was 29 mol % styrene:71 mol % fluoroacrylate.

**Surfactant headgroups – counterion effects:** An important area of investigation is salt addition and counterion exchange in surfactant headgroups. The effects of counterion exchange from Na^+^ to M^2+^ ions ( Mg^2+^, Ca^2+^, Co^2+^, Ni^2+^, Cu^2+^ and Zn^2+^) on Aerosol OT in water-in-oil (*w*/*o*) microemulsions has been investigated [101]. A range of aggregate morphologies was characterised through SANS, with Na(AOT), Mg(AOT)_2_ and Ca(AOT)_2_ forming spherical micelles with reduced viscosities of around 2.5 cm^3^ g^−1^ in comparison to rod-shaped micelles formed by Co(AOT)_2_, Ni(AOT)_2_, Cu(AOT)_2_ and Zn(AOT)_2_ which showed a reduced viscosity of between 10 cm^3^ g^−1^ and 20 cm^3^ g^−1^. Though this was not a CO_2_-water system, AOT has been the basis for many successful CO_2_-philic surfactants, so attempts have been made to apply this strategy to CO_2_ surfactants.

Work using the fluorinated surfactant anion di-HCF4 changing the normal Na^+^ counterion for Co^2+^ or Ni^2+^ resulted in an increase of viscosity between 20–90% over shear rates of 6000–10000 s^−1^ at approximately 6–10 wt % with Ni(di-CF4)_2_ in comparison to the minor viscosity increase seen for the Na^+^ ion analogue (approximately 10% over the same conditions) [49]. The high pressure viscosity data in this study is combined with high pressure SANS (HP-SANS) characterizations which confirmed the presence of anisotropic microemulsion aggregates, or rod-like micelles, within samples containing Ni^2+^ and Co^2+^ counterions. This phenomenon has also been observed with di-CF3 with the replacement of Na^+^ with Ni^2+^ and Co^2+^ counterions [8] but not with the tri-branched hydrocarbon chain TC14 analogue for which spherical aggregates were observed [102].

**Figure 6:**
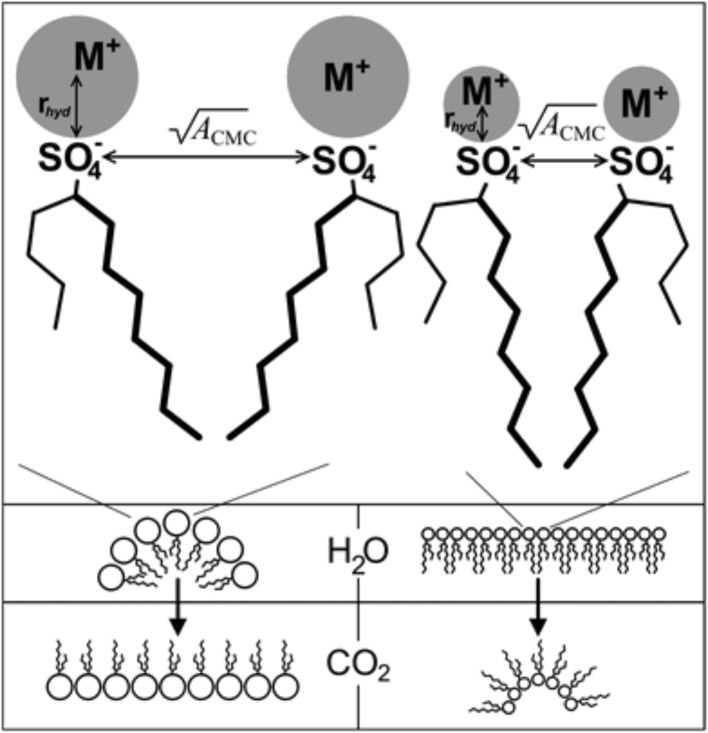
Schematic showing how hydrated cation radius (*r*_hyd_) mediates surfactant headgroup repulsions by controlling limiting headgroup area at critical micelle concentration (*A*_cmc_). Changes in *A*_cmc_ and *r*_hyd_ affect preferred curvature and therefore aggregate morphology in water and scCO_2_. Reprinted with permission from [32]. Copyright 2012 American Chemical Society.

Work around the effects of counterion hydrated radius (*r*_hyd_) and critical packing parameter (CPP) on self-assembled morphologies of the CO_2_-philic hybrid semi-fluorinated surfactant M-F7H4 (pentadecafluoro-5-dodecyl sulfate) was carried out by Cummings et al. through the substitution of a range of metal counterions (Li-F7H4, K-F7H4, Na-F7H4 and Rb-F7H4) [32]. It is known that *r*_hyd_ of the M^+^ ion can impact preferred curvature and therefore impact surfactant morphology, and exchanging counterion induces a change in effective ion size through the hydrated radius (Figure 6).

The study indicated that micelles in water and CO_2_ should have a range of geometric packing parameters as a function of M^+^, as interfacial packing density and limiting the area of the head group at the critical micelle concentration (*A*_cmc_) are dependent on the identity of M^+^ (highlighted through surface tensiometric measurements). Decreases in cmc, *A*_cmc_ and surface tension were observed with a respective increase in the size of the counter ion (Li^+^ < K^+^ < Na^+^ < Rb^+^). *A*_cmc_ is a good approximation of the limiting area per head group in micellar aggregates and can be used to estimate the critical packing parameter, Equation 6.

[6]
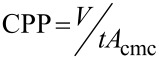


Where *V* is the surfactant tail volume and *t* is the maximum extended length of the tail group chains within the micelle. Surfactants with smaller tail volumes (*V*) and larger head groups (*A*_cmc_) will have CPP values of <1 and will self-assemble with a positive surface curvature, whereas those with smaller headgroups and larger tail volumes will have CPP values of >1, giving rise to a negative surface curvature and reverse micelles. As CPP value of a system decreases towards 1.5–1, reversed cylindrical and rod-like reversed micelles are expected to form.

When CPP ≤ 0.33, normal curved spherical aggregates are expected, if 0.33 < CPP < 0.5, ellipsoidal or rod-like aggregates are expected to form and when 0.5 < CPP < 1, lamellar structures are expected to form. SANS was used to characterise morphologies of M-F7H7 at the air-water interface, which were in line with the predictions from CPP values: Li-F7H4 (CPP = 0.26) and K-F7H4 (CPP = 0.44) formed prolate ellipsoids, Na-F7H4 formed rods (CPP = 0.40) and Rb-F7H4 formed vesicles (CPP = 0.52) at *w* = 12.5 systems. An increase of the *w* value lead to micellar elongation in the Li^+^, Na^+^ and K^+^ systems and no change in the Rb^+^ systems. The area of counterion exchange and its impact on surfactant packing in CO_2_ has been extensively reviewed by James et al. [41]; readers are referred to this for further information on the subject.

**Co-surfactant, salt and additive addition:** Methods of viscosity enhancement in general have been reviewed by Trickett et al. with a specific focus on surfactant gel formation through the production of worm-like micelles [50]. Worm-like micelles have been shown to build viscosity through entangling or cross-linking of the structures, which then generates a structural network and enhance elasticity and viscosity [31,50]. Micellar growth in these systems was induced through the addition of co-surfactants and additives. Though the review does not include gelation in CO_2_ systems, it is still worth mentioning due to the inclusion of fluorocarbon and AOT surfactants since AOT derivatives and fluorocarbon surfactants have both been successfully solubilised in dense CO_2_. The one-dimensional growth of worm-like micelles has been reported with the addition of cosolvents and salts with a range of surfactants, including anionic, cationic and mixed surfactants, in oil-in-water systems [95].

**Figure 7:**
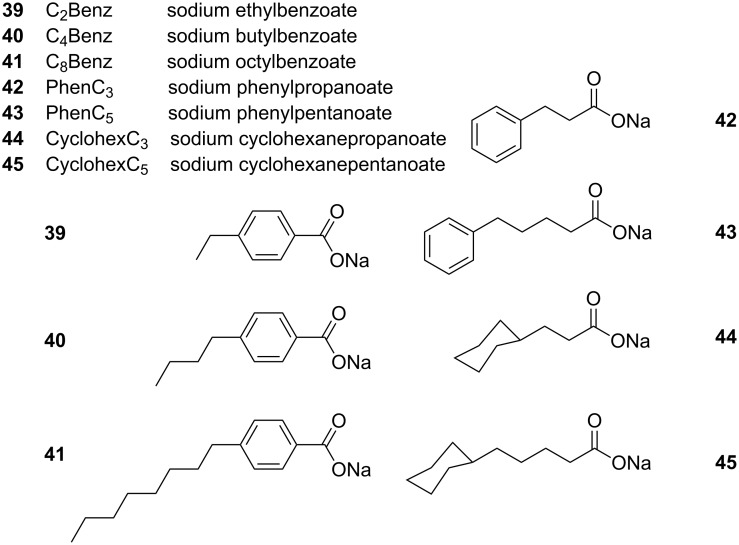
Abbreviated names and structures of hydrotropes tested by Hatzopoulos et al. [40,105,107].

Salt addition to an amphiphilic system is known to screen the charged surfactant headgroup repulsions, which leads to a reduction of the effective headgroup size [103]. This leads to a reduction in preferred curvature (as described by the CPP model) and therefore the formation of microstructures that favour reduced curvature, such as rod-like micelles. Hydrotropic salts have shown to be successful in elongating spherical reverse micellar structures to form rod-like and ellipsoid microemulsions in alkanes, and also interestingly scCO_2_ systems [40,94,104]. Extensive work by Hatzopoulos et al. has been carried out to investigate further the impacts of hydrotropic salt structure and water level (*w*) on AOT surfactant microemulsion structure in *w*/*o* systems [40,105–106]; these findings were used as the basis for designing CO_2_-philic analogues. James et al. studied the impacts of *w* value and hydrotrope concentration in water-in-oil (*w*/*o*) and in water-in-scCO_2_ (*w*/*c*) systems using universal surfactant TC14 [94]. In general, in both *w*/*c* and *w*/*o* systems, as w level is increased morphologies transitioned from cylinders to ellipsoids, and finally to spheres. This is accounted for through the decrease of the aqueous concentration of the hydrotrope toward the critical aggregation concentration (cac) with increased water content; when the hydrotrope concentration decreases below the cac, spherical micelles are formed. In this study, a range of hydrotrope structures was also investigated, see Figure 7. It was also discovered that hydrotropes with longer alkyl tails (compound **40** – C_4_Benz and compound **41** – C_8_Benz) had the greatest solubilisation capacity, followed by the remaining “long” hydrotropes (compound **43** – PhenC_5_ and compound **45** – CyclohexC_5_), which could be due to the destabilizing effects of the rings in the surfactant layers. Hydrotropes with equal numbers of C atoms also had similar upper temperature boundaries (C_4_Benz, C_5_Phen and CyclohexC_5_) [40].

James et al. recently published research around the use of hydrotropes with a universal surfactant TC14 reporting spherical to rod reverse micellar transitions in both *w*/*o* and *w*/*c* systems [94]. Impacts of water content, hydrotrope structure and hydrotrope mole fraction (X, Equation 7) were investigated.

[7]
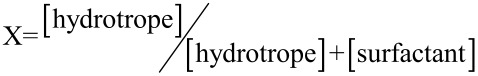


Micellar elongation was observed in water-in-oil systems with increased *w* value; though this is the opposite of that which was observed by Hatzopoulos et al. [40], this is attributed to the aqueous concentration of the hydrotrope never reaching the cac, due to the inability of TC14 to stabilise *w* values as high as those observed within AOT microemulsions [42]. Increased hydrotrope mole fraction also lead to increase in micellar elongation and hydrotrope structure was also shown to impact elongation; however, there was no visible trend around the magnitude of elongation and hydrotrope structure (hydrotrope structures investigated include hydrotropes **39**–**43**, Figure 7). For water-in-CO_2_ systems the structure of the hydrotrope was shown to have minimal impact on morphology and higher mole fractions of hydrotrope gave rise to an increase in microemulsion elongation; this was significantly less pronounced than those seen in water-in-oil systems [94].

**Figure 8:**
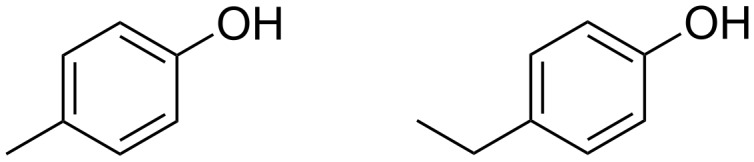
Structures of *para*-methylphenol and *para*-ethylphenol. Para-substituted phenols have been shown to trigger gel formation in AOT w/o systems [94,104] .

AOT surfactant organogels have also been induced in isooctane through the addition of trace levels of para-substituted phenols (*p*-ethylphenol and *p*-methylphenol, Figure 8), with reports of vast viscosity enhancements up to ~5 orders of magnitude with phenol addition being as low as 0.1 mol dm^−3^ [93,104,108]. Particularly stiff gels were formed when the surfactant concentration and the phenol concentration were close to 1:1 and softening appeared when the surfactant:phenol ratio moved to around ≥3:1 or ≤1:3; the gels ‘melted’ when trace amounts of water were added to the system. The gel is thought to be formed through a stacked phenolic structure, with AOT adsorbing onto the external surface. FTIR data indicates that there was hydrogen bonding between AOT and the phenolic species [93,104]. Though AOT is not CO_2_-philic, significant developments surrounding AOT, like those made by John et al. are included in this review, in hope that they may shed light on potential CO_2_ viscosifiers due to the CO_2_-philicity of many AOT analogues.

Hydrotropic salts have also been shown to increase internal structure through intermolecular π–π interactions. Highly ordered macroscale structures were formed through surfactant self-assembly with the addition of the hydrotropic salt benzylamine hydrochloride (BnNH_2_∙HCl) to a sodium sulfonate surfactant (sodium dodecylbenzene sulfonate, SDBS) [109]. With hydrotrope addition, self-assembly of the surfactant into multilamellar vesicles was observed. Multi-lamellar vesicles consequently transform into ultra-long fibres when [BnNH_2_∙HCl] ≥ [SBDS]. These ultra-long fibres are visible through optical microscopy and can entangle which would lead to an increase in viscosity. The one dimensional fibre growth is attributed to directional forces arising from π–π interactions between the phenyl groups present in both the surfactant and the hydrotrope.

#### Conclusions – viscosity enhancers in dense CO_2_

There is a range of approaches to help develop viscosity in scCO_2_, most of which surround the principle of building internal structure. Exchange of surfactant counterion has been known to impact viscosity in both water-in-oil and water-in-CO_2_ systems; research by Eastoe et al. [101] showed that counterion exchange from Na^+^ ions to heavier M^2+^ ions lead to the formation of rod-shaped reverse micelles when transition metal analogues were used as opposed to spherical micelles with the use of Na^+^, Mg^2+^ and Ca^2+^ ions in AOT systems. An increase in reduced viscosity was observed along with the change in morphology. This was then built on through research around counterion exchange in a range of CO_2_-philic surfactants, where the same phenomenon was generally observed and accompanied by a significant viscosity increase when M^+^ ions were exchanged for M^2+^ ions [32,49]. Effective ion size, hydrated ion radius and surfactant critical packing parameters were some of the factors used to explain these phenomena [32,41].

The addition of low molecular weight polymers has shown some promise, with viscosity increases being observed in polymer containing CO_2_ in comparison to pure CO_2_, which are attributed to strong intermolecular forces arising from π–π interactions of phenyl groups in the polymeric constituent [96–100]. Salt addition to systems is also shown to drive the formation of elongated micelles, leading to increased screening of the surfactant headgroups and reduction in effective headgroup size and therefore favouring a reduced surface curvature. Similarly, the addition of hydrotropic salts and phenols to microemulsions can lead to positive viscosity builds in both water-in-oil and water-in-carbon dioxide systems as observed by Eastoe et al. and John et al. [40,94,104]

#### Emerging areas using supercritical CO_2_

**Bicontinuous and lamellar phases, lamellar liquid crystals and foams:** Klostermann et al. has recently reported research around balanced supercritical CO_2_ microemulsions; systems where there are equal volumes of carbon dioxide and water [43]. These systems use a polyfluoroether surfactant (poly(ethylene glycol) perfluoroalkyl ether), along with commercially available ethoxylated surfactants Zonyl FSN 100 and Zonyl FSO 100 and NaCl. SANS data and phase behaviour work indicated that these systems follow a bicontinuous structure, with microemulsion domain size increasing with increased pressure [43].

Sagisaka et al. reported the production of water-in-CO_2_ lamellar structures after a study using fluorinated double-chain anionic surfactants with varied chain lengths. Surfactants with chain length *n* = 4, 6, & 8 were found to form spherical reverse micelles and those with chain length *n* = 2 were found to form lamellar structures (confirmed through SANS). These observations are accounted for through the reduction of CPP with shorter fluorocarbon surfactant tails. The surfactant where *n* = 4 showed the highest solubilizing power out of those tested (*w* = 80); the reason for this is still unclear, although it has been proposed that this is due to the formation of a water-in-CO_2_ bicontinuous microemulsion (also based of the reduction of CPP value) [48].

Carbon dioxide-in-water (*c*/*w*) foams have been formed with a range of branched non-ionic hydrocarbon surfactants and their viscosities, stabilities and morphologies studied using microscopy and capillary microscopy for potential application in CCS and EOR [110–111]. Surfactant design requirements are more flexible for *c*/*w* foams in comparison than for *w*/*c* microemulsions, due to the lower γ values needed to stabilise *w*/*c* microemulsions [112]. Branching in the surfactant tails was seen to increase foam stability in comparison to linear chain surfactants, through reducing the contact of water and CO_2_. The greater stability of *c*/*w* films in comparison to air-in-water (*a*/*w*) was attributed to a smaller film size as well as smaller γ and π (surface pressure) values.

**Figure 9:**
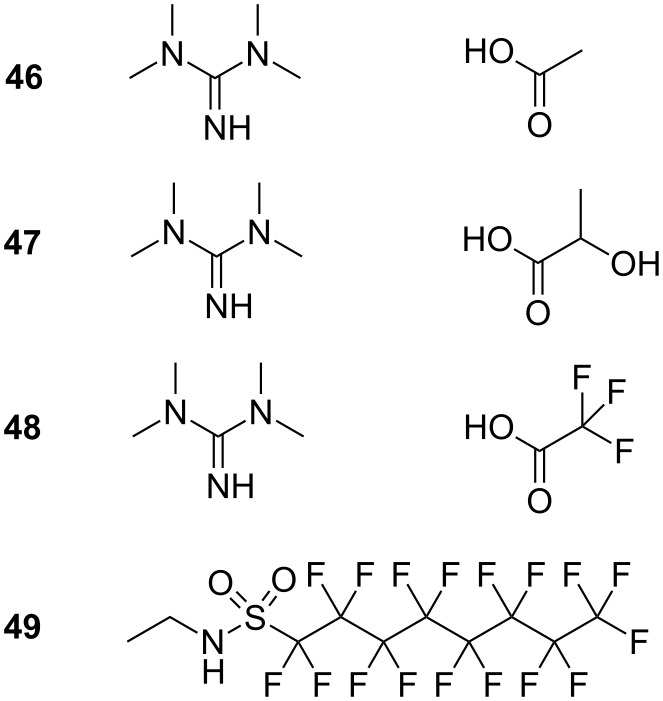
Structures of 1,1,3,3-tetramethylguanidinium acetate (**46**), 1,1,3,3-tetramethylguanidinium lactate (**47**), 1,1,3,3-tetramethylguanidinium trifluoroacetate (**48**) and *N*-ethyl perfluorooctylsulfonamide (**49**), as used in IL-in-CO_2_ microemulsions.

**Ionic liquid-in-scCO_2_:** Room temperature ionic liquids (ILs) are organic salts composed purely of organic and inorganic ions with a melting point below 100 °C. They have recently received a significant amount of attention, and due to their chemical stability, non-flammability, low toxicity and low volatility are, alongside supercritical CO_2_, regarded as green solvents [113]. Research has particularly focused around combining ILs with scCO_2_ to form microemulsions with the hope to combine the advantages of each solvent. Liu et al. have reported the formation of IL-in-CO_2_ microemulsions using a highly fluorinated surfactant, *N*-ethyl perfluorooctylsulfonamide (compound **49**, Figure 9). Ionic liquids solubilised included 1,1,3,3-tetramethylguanidinium acetate (TMGA, compound **46**, Figure 9), 1,1,3,3-tetramethylguanidinium lactate (TMGL, compound **47**, Figure 9) and 1,1,3,3-tetramethylguanidinium trifluoroacetate (TMGT, compound **48**, Figure 9). Additives such as methyl orange, CoCl_2_ and HAuCl_4_ were solubilised within the IL domains, which were shown to be spherical micelles by transmission electron microscopy (TEM). The group also observed an increase in *P*_trans_ with increased *w* value, as expected (*w* = 0.1–0.8) [54].

Chandran et al. have mapped the formation of reverse IL micelles in scCO_2_ through a computer simulation technique, which also gives evidence for the production of stable IL droplets within a continuous CO_2_ phase through amphiphilic surfactant addition. This study suggests that microemulsion stability is dependent on ionic liquid anion-surfactant headgroup interactions and that ionic liquid cations play only a minor role [55]. The study was also indicative of the presence of ellipsoidal reverse micelles, which is supported by SANS data previously carried out on a similar IL-in-oil system [56].

## Conclusion

Over the last 20 years there have been significant developments surrounding the use of supercritical carbon dioxide, both as an alternative green solvent to volatile organic compounds but also towards the efficiency of carbon dioxide handling with a view to enhance carbon capture and sequestration techniques, as well as enhanced oil recovery. This has come in several forms, through identifying factors that lead to an enhancement of CO_2_-philicity in additives as well as the development of CO_2_ viscosifiers. A combination of these breakthroughs may lead to both commercially viable and practical surfactants and additives that will effectively thicken CO_2_. A myriad of structures has been observed in water-in-CO_2_ systems, including a range of reverse micellar shapes to bicontinuous phases and lamellar structures. The virtuous properties of scCO_2_ as a solvent could be enhanced further through the use of IL-in-CO_2_ systems, thus combining with the positive properties of ILs have as solvents. Though noteworthy breakthroughs have been made around this area, more work needs to be undertaken to overcome the uncooperative nature of CO_2_ to fully utilize it as a solvent and processing medium.
